# Spread of Influenza A(H1N1) oseltamivir-resistant viruses in Africa in 2008 confirmed by multiple introductions in Senegal

**DOI:** 10.1186/1471-2334-13-106

**Published:** 2013-02-27

**Authors:** Ndongo Dia, Mbayame N Niang, Saadiya A Diadhiou, Déborah G Goudiaby, Abdourahmane Faye, Davy Kiori, Mady Bâ, Rémy Michel, Ousmane M Diop

**Affiliations:** 1 Institut Pasteur de Dakar, Unité de Virologie Médicale, Dakar, Sénégal; 2 Ministère de la Santé et de la Prévention, Dakar, Sénégal; 3 Institut Pasteur de Dakar, Unité d’Epidémiologie des maladies infectieuses, Dakar, Sénégal; 4 Unit of Medical Virology, Institut Pasteur de Dakar, Dakar, BP 220, Senegal

**Keywords:** Influenza, Oseltamivir, Resistance

## Abstract

**Background:**

Among Influenza neuraminidase inhibitors (NAIs), oseltamivir corresponds to the most widely used agent to treat influenza disease. However since 2001, several cases of resistance to NAIs have been reported for circulating seasonal A(H1N1) Influenza viruses. A direct resistance mechanism may be invoked, involving critical mutations in the viral NA gene that prevent the drug binding to its target. Same phenomenon is reported for adamantanes drugs and mutations in the M2 channel protein gene of Influenza viruses.

**Methods:**

Reverse-Transcription/Restriction Fragment Length Polymorphism (RT-PCR/RFLP) method, phenotypic testing for oseltamivir resistance, and sequencing of NA, HA and M2 genes were used in this study. Phylogenetic analyses were performed using BioEdit and Mega 5 softwares for alignment of sequences and phylogenetic trees building respectively.

**Results:**

Using a simple RT-PCR/RFLP method, we found that the 86 seasonal A(H1N1) isolates from 2008 bear the oseltamivir resistance-associated mutation (H274Y) in the NA gene. In contrast all isolates isolated in Senegal in 2007 were sensitive to oseltamivir. These results were first confirmed by finding high IC50 values using a phenotypic testing for oseltamivir resistance, and secondly by sequencing the whole NA gene. Regarding M2 gene, no mutation associated to adamantanes resistance was characterized of the isolates.

**Conclusions:**

The present work provides evidence of circulation of drug-resistant seasonal A(H1N1) viruses during the 2008 influenza season (July to September) in Senegal. The results are in favor of multiple introductions of oseltamivir resistant viruses (ORV) A(H1N1) in Senegal.

Phylogenetic analyses of isolates with complete sequences of N1 and HA1 genes showed that they belong to clade 2B and suggest sequential introductions in Africa.

## Background

An important progress in the treatment of influenza during the past decade has been the development of potent, selective inhibitors of the viral neuraminidase (NA) enzyme. This was especially achieved through the structure-based design of carboxylic sialic acid analogues interacting with the NA active site, a highly conserved site in both influenza A and B viruses [1,2]. Among these NA inhibitors (NAIs), oseltamivir corresponds to the most widely used agent to treat influenza disease. However since 2001, sporadic cases of resistance to NAIs have been reported [3,4]. A direct resistance mechanism may be invoked, involving some mutations in the viral NA gene that prevent the drug binding to its target [5]. The relation between oseltamivir resistance and histidine-to-tyrosine mutation at the position 274 (275 in N1 numbering system) was clearly established [6].

The major event of the 2007-2008 influenza season in Europe was the emergence of oseltamivir-resistant seasonal A(H1N1) viruses. Indeed, by the end of January 2008, the emergence of influenza A(H1N1) oseltamivir-resistant viruses (ORV) was first reported in Norway [7,8], and rapidly in many other European countries [9,10]. The overall frequency of oseltamivir resistance in A(H1N1) viruses from Europe was 25%, with variation between countries; Norway detecting the highest proportion (67%), and others have detected proportions as low as 2%, e.g in Spain [9]. Following the emergence of this resistant strain in Europe, these variants were also reported in the southern hemisphere [11]. With regard to Africa, the first surveillance data on oseltamivir resistance came from South Africa and showed that all viruses isolated in 2008 were resistant to oseltamivir, on the basis of ARMS PCR results [12]. Given that the predominant subtype of influenza virus circulating in Senegal during the 2008 season (from July to October) was seasonal A(H1N1), we used these isolates to test whether the spread of oseltamivir-resistant variants may have reached countries in the inter-tropical areas of Africa.

Two adamantane derivatives, amantadine and rimantadine namely, are also used as antiviral drugs for prophylaxis and treatment of influenza. These drugs bind to and block the function of the influenza A virus M2 ion channel protein, preventing virus replication within the infected cell [13]. So resistance to adamantanes can emerge during treatment. A single point mutation in the sequence coding for the amino acid at position 26, 27, 30, 31, or 34 of the M2 protein confers resistance to the adamantanes [14,15]. In this study, in addition to oseltamivir resistance, our aim is also to look for these genetic markers for adamantane resistance by sequencing the critical region of the M2 gene harboring these different amino acid positions.

It is worth noting that no influenza antiviral agents, including oseltamivir, amantadine or rimantadine, were used in Senegal.

## Methods

Specimens were collected from subjects with influenza-like illnesses as part of the sentinel surveillance system for Influenza-like Illness (ILI) conducted in Dakar since 1996 and as requested by the Ministry of Health in order to decipher the etiology of febrile illnesses in children seeking care in primary healthcare centers in Dakar.

An ILI case was identified as an outpatient presenting with sudden onset of fever (≥38°C) and cough or sore throat, accompanied or not by myalgia, prostration, headache or malaise, with the onset of symptoms occurring within the previous 3 days. A standardized questionnaire was used to collect demographic and clinical information from the enrolled patients. Throat and/or nasopharyngeal swabs were taken from patients within 48–72 hours from onset of symptoms and then tested for virus isolation and/or molecular detection with standard protocols validated by WHO. When both throat and nasal swabs were available, the two samples were collected in the same tube to increase sensitivity of testing. Aliquots of samples were also stored at -80°C for further analysis.

For virus isolation, the clinical materials obtained from patients were inoculated on Madin Darby Canine Kidney (MDCK) cell lines as described previously [16]. Tissue culture fluid was harvested after observing inoculated MDCK cell line for cytopathic effect. Hemagglutination and Hemagglutination Inhibition (HAI) tests were performed (detection and typing of viruses) using specific antisera, as recommended by WHO standard protocols [17].

For molecular characterizations, ribonucleic acid (RNA) extraction was performed from 200 μl of each sample using the QIAamp Viral RNA kit (QIAGEN, Valencia, CA, USA) according to the manufacturer’s instructions. Each RNA sample was eluted with 50 μl of nuclease-free water.

We used an RT-PCR/RFLP technical [18] to discriminate between oseltamivir-sensitive and oseltamivir-resistant influenza A(H1N1) isolates. RT-PCR primers target a 183-bp region of the NA gene, encompassing the codon prone to H274Y mutation (N1-770Fw: AGA TCG AAA AGG GGA AGG TTA CTA, and N1-881Rv: TCC CTG CAT ACA CAC ATC ACT). Briefly, 6 μl of each RT-PCR product were digested with BspHI (New England Biolabs®) in a final volume of 20 μl, the restriction site for this enzyme (T**CAT**GA/AGTACT palindrome) covering the codon 274 (H: **C**AT/Y: **T**AT). Thus, BspHI should cut only amplicons without mutation, *i*.*e*. amplicons from oseltamivir-sensitive A(H1N1) viruses (predicted size of the major digest product: 147 bp). Digested and undigested amplicons were co-electrophoresed on 2% agarose with appropriated molecular weight markers (100 bp ladder, New England Biolabs®). Oseltamivir-sensitive isolates from the 2007 Influenza season served as positive controls for BspHI digestion. Gels were stained with ethidium bromide (0.5 μg/ml).

The matrix M2 gene was amplified and sequenced in order to look for mutations conferring resistance to adamantanes.

Selected viruses were phenotypically (resistance to NAI), antigenically, and genetically characterized by the WHO Collaborating Centre for Influenza at the National Institute for Medical Research (NIMR) in London, UK. The influenza virus neuraminidase (NA) activity of the isolates and sensitivity to neuraminidase inhibitors (NI) was measured by an enzyme assay using a fluorogenic substrate i.e MUNANA (2’ 2^′^-(4-Methylumbelliferyl)-α-D-N-acetylneuraminic acid sodium salt hydrate) with known NA inhibitor-resistant viruses as controls. HA and NA genes were sequenced using ABI Prism BigDye terminator cycle sequencing kits and an ABI-3730XL DNA analyser. Phylogenetic analysis of the neuraminidase whole gene sequence (1400 bp) and the haemagglutinin HA1 genes (1200 first bp) of H1N1 viruses were performed using MEGA version 5 [19] for constructing Maximum Likelihood Tree using the Tamura-nei evolutionary model. Alignments of sequences were performed using the BioEdit Sequence alignment Editor [20].

The surveillance protocol was approved as less than minimal risk research by the Senegalese National Ethical Committee of the Ministry of Health, and written consent forms were not required. Throughout the study, the database was shared with the Epidemiology Department at the Senegalese Ministry of Health and Prevention for appropriate public health actions.

## Results

A total of 86 H1N1 viruses were isolated from July 2008 (week 28) to September 2008 (week 38). The median age of patients tested positive for viral isolation was 5 years (range: from 2 months to 48 years), 66% of these patients being young children (no more than 2 years) and most of them (74%) living in the suburbs of Dakar.

**Figure 1:**
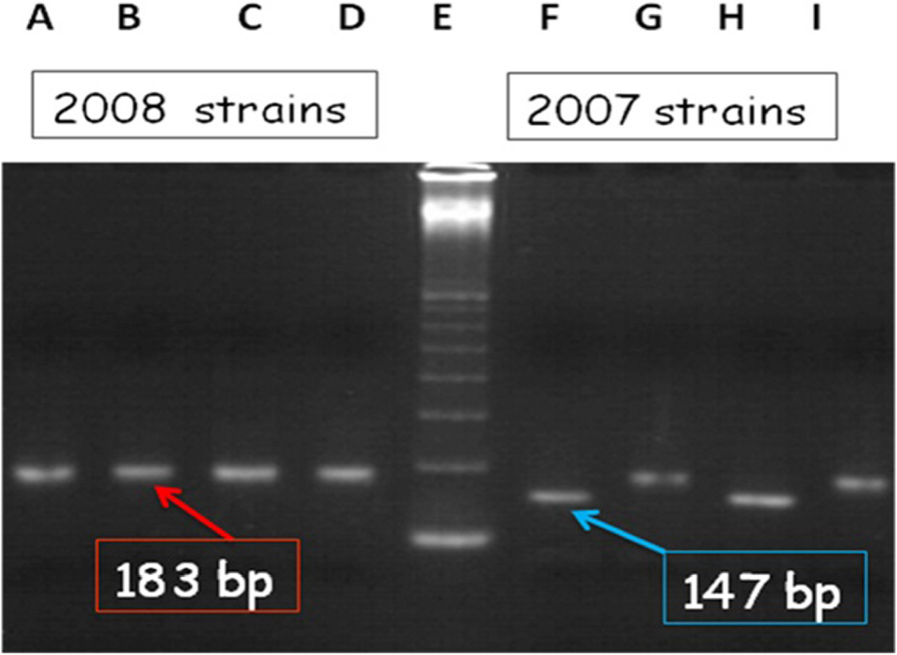
**RT**-**PCR**/**RFLP analysis of the NA gene of seasonal A(H1N1) influenza viruses digestion with *Bsp*HI enzyme of DNA amplicons obtained by RT**-**PCR targeting the neuraminidase gene of influenza A(H1N1) isolates and covering the mutation site conferring oseltamivir resistance.** Tracks A to D: A(H1N1) viruses isolated in 2008; Tracks F to I: A(H1N1) viruses isolated in 2007; Tracks A, C, F, H: amplicons digested with *Bsp*HI, Tracks B, D, G, I: undigested PCR amplicons. Track E: 100-base pair marker (New England BioLabs).

The data showed that PCR products from all 86 isolates of the 2008 season are not digested by the restriction enzyme BspHI in contrast to the 2007 isolates (Figure 1). This suggests a high frequency of the H274Y mutation in the N1 gene and therefore a potential resistance to oseltamivir of the studied viruses. The emergence of oseltamivir-resistant viruses in 2008 was confirmed by the finding of a high IC50 mean value (859.3 nM) compared to the one obtained for 2007 viruses (between 1 and 2 nM) (Table 1). In 2007, all the 27 A/(H1N1) strains sent to the WHO Collaborating Centre for Influenza in NIMR, London, were sensitive to Oseltamivir and didn’t harbor the H274Y mutation.

**Table 1:** **Phenotypes of seasonal influenza A**(**H1N1**) **viruses isolated in Dakar**, **Senegal**, **in 2008**

**Seasonal A/H1N1 viruses**	**Oseltamivir IC50 (nM)**	Phenotype (S/R)*
A/Dakar/03/08	1032.4	R
A/Dakar/04/08	943.6	R
A/Dakar/05/08	577.7	R
A/Dakar/06/08	517.5	R
A/Dakar/07/08	430.5	R
A/Dakar/09/08	545.5	R
A/Dakar/11/08	1115.3	R
A/Dakar/12/08	999.6	R
A/Dakar/13/08	1042.9	R
A/Dakar/14/08	775.0	R
A/Dakar/2007	1-2	S

_*_S = Sensitive; *R* = Resistant.

Results of testing sensitivity to oseltamivir.

Phenotypic resistance to oseltamivir for seasonal influenza A(H1N1) viruses isolated in 2008 is compared with isolates obtained in 2007, in Dakar, Senegal. The mean value of the Inhibition Concentration 50 for 2008 isolates is 859.3nM (range from 430.5 to 1115.3).

Ten (10) isolates were tested for resistance to adamantanes and oseltamivir through sequencing of the matrix M2 gene and the N1 portion that recover the H275Y mutation. None of the five mutations conferring resistance to adamantanes [21] were detected in contrast all isolates harbored the H275Y mutation conferring oseltamivir resistance.

**Figure 2:**
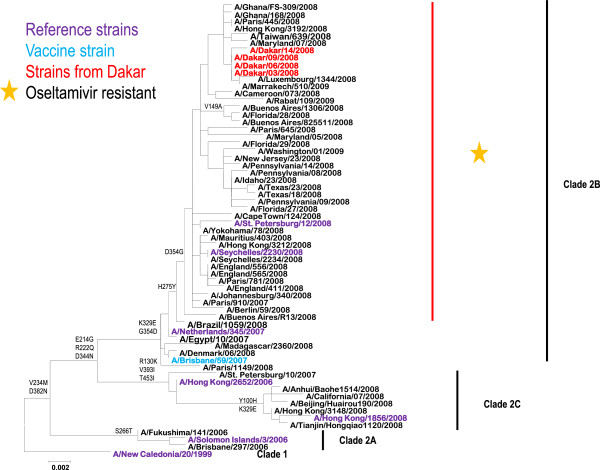
**Phylogeny of of the NA gene of seasonal A(H1N1) influenza viruses.** Phylogenetic analysis of the neuraminadase gene sequences of H1N1 viruses, using MEGA version 5 for constructing Maximum Likelihood Tree using the Tamura-nei evolutionary model. The prototype reference strains for each clade are colored in purple, the contemporary vaccine virus recommended for inclusion of the trivalent influenza vaccine for the 2008/2009 Northern Hemisphere winter is shown in blue ; Viruses from Dakar, Senegal, are shown in red. The group indicated by the asterix carries the H275Y amino acid substitution.

The N1 gene of the four (4) isolates (GISAID identification numbers: A/Dakar/03/2008/EPI_ISL_65453, A/Dakar/06/2008/EPI_ISL_65454, A/Dakar/09/2008/EPI_ISL_65455 and A/Dakar/14/2008/EPI_ISL_65456) for which sequencing were done in a WHO Collaborative Center (NIMR, London, UK) were then analyzed along with published N1 sequences (Figure 2). As expected, all Dakar isolates were in a sub-clade carrying the H275Y mutation.

**Figure 3:**
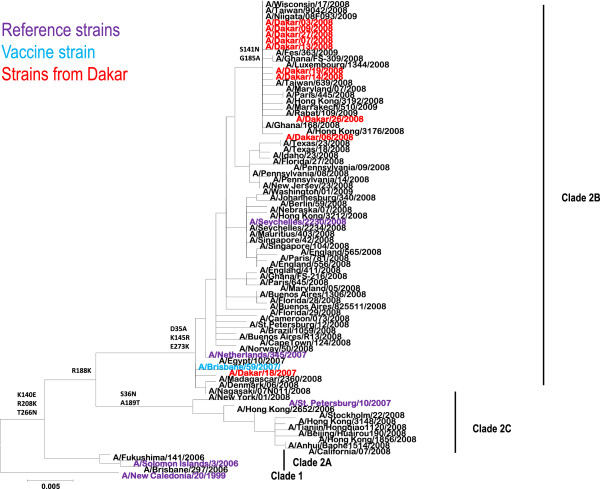
**Phylogeny of HA1 segment of the HA gene of seasonal A(H1N1) influenza viruses.** Phylogenetic analysis of the neuraminadase gene sequences of H1N1 viruses, using MEGA version 5 for constructing Maximum Likelihood Tree using the Tamura-nei evolutionary model. The prototype reference strains for each clade are colored in purple, the contemporary vaccine virus recommended for inclusion of the trivalent influenza vaccine for the 2008/2009 Northern Hemisphere winter is shown in blue ; Viruses from Dakar, Senegal, are shown in red.

To evaluate the genetic heterogeneity of Dakar isolates, we undertook a phylogenetic analysis based on the HA1 portion of the hemagglutinin (HA) gene. HA1 sequencing was performed in five randomly selected isolates (GISAID identification numbers: A/Dakar/07/2008/EPI_ISL_117092;A/Dakar/13/2008/EPI_ISL_117094;A/Dakar/19/2008/EPI_ISL_117095, A/Dakar/26/2008/EPI_ISL_117096 and A/Dakar/27/2008/EPI_ISL_117097). Five additional isolates (GISAID identification numbers: A/Dakar/03/2008/EPI_ISL_65453; A/Dakar/06/2008/EPI_ISL_65454; A/Dakar/09/2008/EPI_ISL_65455; A/Dakar/14/2008/EPI_ISL_65456 and A/Dakar/18/07/EPI_ISL_18609) were sequenced in NIMR, WHO CC, London and included in this study. No systematic geographic clustering of the viruses collected from different continents (Africa, Europe and North America) is evident as shown in the phylogenetic tree (Figure 3). So all 2008 viruses isolated in Dakar, Senegal, are distributed into a well-distinct group. These isolates cluster in a diverging sub-clade, characterized by two characteristic mutations in the HA1 segment compared to the other isolates (S141N and G185A). In this sub-clade are also represented isolates from Europe, Asia, North America and others African countries. The remaining isolate from Dakar (A/Dakar/18/07, isolated in 2007) belong to a different group which comprise the A/Brisbane/59/2007 vaccine virus. These results are strongly in favor of multiple introductions of A(H1N1) ORV in Senegal in 2008.

## Discussion

In this paper sequencing of Influenza NA genes has validated utilization of RT-PCR-RFLP method for direct detection of oseltamivir-resistant influenza viruses, with a 100% concordance between the two methods. This method will then be a very useful tool for laboratories without direct access to sequencing facilities, as many laboratories in poor resource settings, including Africa. The present work also provides the first evidence of circulation of drug-resistant A(H1N1) viruses during the 2008 influenza season (July to September) in Senegal after years of flu monitoring [22], as previously reported for South Africa [12]. These results are in favor of multiple introductions of oseltamivir resistant viruses (ORV) A(H1N1) in Senegal, rather than a re-emergence of previously circulating viruses that developed resistance to oseltamivir. Indeed all influenza A(H1N1) viruses in 2007 in Senegal were sensitive to Oseltamivir and this drug has not been used in Senegal to treat patients infected by influenza virus. Even if some studies supported that oseltamivir resistance is low in patients treated with oseltamivir [23], two previous studies in Japan revealed that oseltamivir resistance emerged in 18% and 16% of treated Japanese children with influenza virus A(H3N2) and A(H1N1) infection respectively [24,25]. Moreover, prescription data for NAI treatment indicate that these drugs are not widely used in Europe despite the high rate of spread in a short time [11]; by contrast, in Japan just during the 2003–04 season, ≈6 million NAI treatment courses were prescribed [26].

The close relationships of the viruses isolated in Dakar with those from Europe, North America, Asia and other African countries, as shown by phylogenetic analyses, may signal virus’ introduction(s) in Dakar from these areas. Indeed the air traffic between Dakar and these countries is quite dense. However, it is more probable that the spread of oseltamivir-resistant influenza A(H1N1) viruses may have followed a path from South Africa northwards to the Sahelian regions of Africa. To substantiate this hypothesis, the phylogenetic tree based on the HA1 gene (Figure 3) show a genetic/antigenic drift (in comparison to the 2007 reference and vaccine strains) more marked for viruses from West Africa (Ghana and from Senegal) than for viruses from Southern and Central Africa. Strains isolated in Senegal in 2008 are exclusively located in a group excluding viruses from South Africa and Cameroon. Moreover, it is well established that South Africa is the first country in Africa that reported ORV cases [12], three months before detection of such cases in Senegal. In addition, RT-PCR/RFLP analysis of influenza A(H1N1)viruses detected in Mauritania (located in the north of Senegal) in December 2008 showed that they are resistant to oseltamivir and closely related to viruses that have circulated in Senegal three to four months ago (data not shown). Thus the South-to-North spread of drug-resistant influenza viruses in Africa seems likely even if one cannot exclude some direct seeding from other regions. Even though analyses of influenza viruses bearing the H274Y marker conferring resistance to oseltamivir provides some clues, further studies are needed to decipher the complex dynamics of Influenza in the African continent. Indeed, (i) the lack of data on the month of isolation of some strains included in the phylogenetic tree, and (ii) the intrinsic limitation of the surveillance systems to rapidly detect introduction of influenza strains and discrimination of infections acquired within or outside the country, cannot allow proper tracking of the timeline of introduction of these oseltamivir resistant viruses in each country. On an another hand, even if the travel history of the enrolled patients was not always known it is probable that infections occurred within the country as the study population is sedentary and the majority (66%) of enrolled patients is children less than 2 years old.

## Conclusions

The present study provides genetic evidence of circulation of drug-resistant seasonal A(H1N1) viruses in Senegal during the 2008 influenza season (July to September). It shows that RT-PCR-RFLP method, a simple, reliable and cheap tool can be useful for low-resources’ laboratories to detect circulation of oseltamivir-resistant A(H1N1) viruses. Moreover, the results are in favor of multiple introductions of oseltamivir resistant viruses (ORV) A(H1N1) in Senegal. Phylogenetic analyses of these isolates with complete sequences of N1 and HA1 genes from other continents and areas in Africa showed that they belong to clade 2B and suggest sequential introductions and internal dynamics in Africa during the 2008-2009 Influenza season.

## Abbreviations

ARMS PCR: amplification refractory mutation system PCR; HA: Hemmagglutinin; IC50: 50% maximum inhibitory concentration; ILI: Influenza-like Illness; M2: Matrix 2 gene; MDCK: Madin Darby Canine Kidney; NA (N1): Neuraminadase; NAIs: NA inhibitors; RFLP: Restriction Fragment Length Polymorphism; RT-PCR: Reverse Transcription Polymerase Chain Reaction; WHO: World Health Organization

## Competing interests

The authors declare that they have no competing interests.

## Authors’ contributions

The work presented here was carried out in collaboration between all authors. *OMD* defined the research, analyzed and interpreted the data, and revised the manuscript; *ND* and *MNN* performed and coordinated technical work; ND wrote the draft and revisions of the paper; *DGG*, *DK and AF* contributed in the technical part of this work; *RM* participated in the monitoring of the surveillance sites; *SAD and MB*, are from the Ministry of Health and have coordinated the influenza disease monitoring in Senegal in 2008. All authors contributed to, have seen and approved the manuscript.

## Pre-publication history

The pre-publication history for this paper can be accessed here:

http://www.biomedcentral.com/1471-2334/13/106/prepub
